# 909. Impact of Implementing an IV to PO Antibiotic Treatment Protocol for Orthopedic Infections on Prescribing Habits and Health Utilization Outcomes

**DOI:** 10.1093/ofid/ofac492.754

**Published:** 2022-12-15

**Authors:** Chanah Gallagher, Russell J Benefield, Laura Certain

**Affiliations:** University of Utah Health, Salt Lake City, Utah; University of Utah Health, Salt Lake City, Utah; University of Utah, Salt Lake City, Utah

## Abstract

**Background:**

The Oral versus Intravenous Antibiotics for Bone and Joint Infection Trial concluded that oral antibiotics administered during the first six weeks of therapy for orthopedic infections were non-inferior to parenteral antibiotics. Previously, we also conducted a retrospective cohort study of patients at our center with an orthopedic infection. Patients transitioned to oral therapy had significantly fewer adverse events and similar incidences of one year treatment failure compared to patients maintained on parenteral vancomycin.

**Figure:**
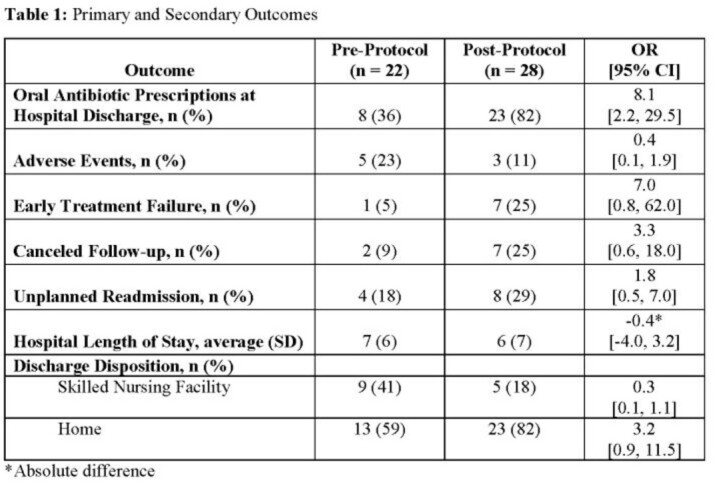

**Methods:**

To increase use of oral antibiotics for orthopedic infections, we created a protocol for changing IV to PO antibiotics prior to hospital discharge. We evaluated its effect on prescribing practices and healthcare utilization outcomes. Patients with an orthopedic infection were included if they were discharged within the 90-day pre- and post-protocol study periods, had an inpatient Infectious Disease consult, and were initiated on parenteral vancomycin while inpatient. The primary outcome compared the incidence of oral antibiotics prescribed at discharge. Secondary outcomes included incidence of adverse drug reactions, discharge dispositions, hospital length of stay, canceled follow-up appointments, unplanned readmissions, and early treatment failure.

**Results:**

Fifty patients were included, 22 and 28 patients in the pre- and post-protocol groups, respectively. Twenty-three (82%) patients in the post-protocol group were prescribed oral antibiotics at discharge compared to 8 (36%) patients in the pre-protocol group. Twenty-three (82%) patients in the post-protocol group were discharged home compared to 13 (59%) patients in the pre-protocol group. Of note, 7 (25%) patients in the post-protocol group had early treatment failure compared to 1 (5%) patient in the pre-protocol group, and 8 (29%) patients in the post-protocol group compared to 4 (18%) patients in the pre-protocol group had an unplanned readmission.

**Conclusion:**

Providing physicians with an oral antibiotic protocol for orthopedic infections significantly increased the number of oral antibiotic prescriptions at hospital discharge, but the higher rates of treatment failure observed after protocol implementation are concerning and warrant further investigation.

**Disclosures:**

**Russell J. Benefield, PharmD, BCPS-AQ ID**, Paratek Pharmaceuticals: Grant/Research Support.

